# Genome-wide DNA methylation profile in mungbean

**DOI:** 10.1038/srep40503

**Published:** 2017-01-13

**Authors:** Yang Jae Kang, Ahra Bae, Sangrea Shim, Taeyoung Lee, Jayern Lee, Dani Satyawan, Moon Young Kim, Suk-Ha Lee

**Affiliations:** 1 Department of Plant Science and Research Institute of Agriculture and Life Sciences, College of Agriculture and Life Sciences, Seoul National University, Seoul 151-921, Korea; 2 Indonesian Center for Agricultural Biotechnology and Genetic Resources Research and Development, Bogor 16111, Indonesia; 3 Plant Genomics and Breeding Institute, Seoul National University, Seoul, 151-921, Korea

## Abstract

DNA methylation on cytosine residues is known to affect gene expression and is potentially responsible for the phenotypic variations among different crop cultivars. Here, we present the whole-genome DNA methylation profiles and assess the potential effects of single nucleotide polymorphisms (SNPs) for two mungbean cultivars, Sunhwanogdu (VC1973A) and Kyunggijaerae#5 (V2984). By measuring the DNA methylation levels in leaf tissue with the bisulfite sequencing (BSseq) approach, we show both the frequencies of the various types of DNA methylation and the distribution of weighted gene methylation levels. SNPs that cause nucleotide changes from/to CHH – where C is cytosine and H is any other nucleotide – were found to affect DNA methylation status in VC1973A and V2984. In order to better understand the correlation between gene expression and DNA methylation levels, we surveyed gene expression in leaf tissues of VC1973A and V2984 using RNAseq. Transcript expressions of paralogous genes were controlled by DNA methylation within the VC1973A genome. Moreover, genes that were differentially expressed between the two cultivars showed distinct DNA methylation patterns. Our mungbean genome-wide methylation profiles will be valuable resources for understanding the phenotypic variations between different cultivars, as well as for molecular breeding.

Mungbean (*Vigna radiata* [L.]) is a self-pollinated diploid plant with 11 chromosomes (2n = 22), which taxonomically, belongs to the Phaseoleae tribe and the Fabaceae family. It is an important legume crop that is widely cultivated in Asia and serves both as a cash crop and as an important source of nutrition. The mungbean can be used in several ways; the seeds, sprouts, and young pods are all consumed as sources of protein, amino acids, vitamins, and minerals, and its by-products are also used as green manure and feed^1^. Thus, because of its importance, the whole draft genome of mungbean was recently constructed, providing a rich genetic resource for researches that will facilitate mungbean breeding^2^.

DNA methylation is an epigenetic modification that influences transposon silencing and gene regulation^3^,^4^. Thus, the polymorphism resulting from differential DNA methylation is an additional factor that, together with nucleotide variation, can contribute to phenotypic variation^5^. In soybeans, epigenetic variations in the DNA were detected at the whole-genome level, and, with few exceptions, co-segregated with nucleotide variations in recombinant inbred lines, suggesting that DNA methylation should be considered in molecular breeding^6^. In this case, DNA methylation occurred on cytosine residues in the following contexts within the genome: CG, CHG, and CHH, where H represents A, T, or C. The maintenance of the modifications at CG, CHG, and CHH were reported to be under the control of different pathways. Specifically, the activities of methyltransferase 1 (MET1) and chromomethylase 3 (CMT3) were responsible for the CG and CHG methylations. Domain rearranged methyltransferase 1 and 2 (DRM1 and DRM2), which are guided by 24 nt small interfering RNAs, are responsible for CHH methylation^7^,^8^. The specificity of these maintenance pathways suggests that nucleotide variations affecting cytosine context would change the methylation maintenance. This possible dependence of DNA methylation on nucleotide variations has been proposed as obligate epialleles^9^,^10^.

With the recent advances in sequencing technologies, along with the availability of fine-tuned chemistries, obtaining the whole-genome DNA methylation profile is feasible *via* a method known as bisulfite sequencing (BSseq)^11^. Treatment of genomic DNA with sodium bisulfite converts the cytosines into uracils; however, methylcytosine will not be converted. Thus, by treating the sequencing libraries with sodium bisulfite, we can detect the status of DNA methylation at the single nucleotide level with next generation sequencing (NGS). In this study, we applied the BSseq method to genomic DNA extracted from the leaf tissues of two mungbean genotypes, VC1973A and V2984. The gene expression levels in leaf tissues were also measured by RNAseq. Using this strategy, we were able to successfully profile the DNA methylation status of the mungbean genome and compared the methylation patterns of the VC1973A and V2984 cultivars to identify possible obligate epialleles that may serve as important genetic markers for breeding. Additionally, we found cases where genes that were differentially expressed in the two cultivars also contained distinct DNA methylation patterns. These results will be valuable for unraveling the role of DNA methylation in plant gene expression and will also serve as an important genomic resource for understanding the phenotypic variations of mungbean cultivars for breeding.

## Results

### Whole methylome and transcriptome sequencing of mungbean

**Figure 1:**
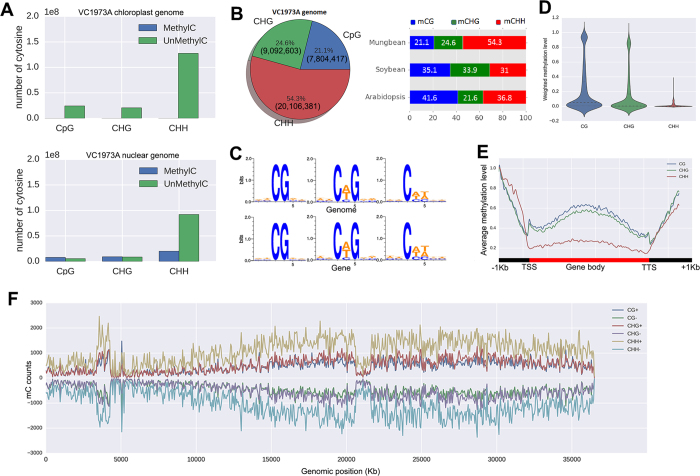
DNA methylation profiles of mungbean genomes, VC1973A and V2984. (**A**) The number of cytosines in the methylated and unmethylated state for the nuclear and chloroplast genome. (**B**) The proportion of mCGs, mCHGs, and mCHHs in the VC1973A genome. (**C**) DNA sequence logo plot of the methylated cytosine contexts. (**D**) Density plots of the weighted methylation levels for mCG, mCHG, and mCHH. (**E**) Average methylation levels for gene bodies and their flanking regions. (**F**) The distribution of methylated cytosine counts in 50 Kb windows of chromosome 1.

To generate a DNA methylation map at the single base resolution across the mungbean genome, whole-genome bisulfite sequencing (BSseq or MethylC-seq)^3^ was performed on genomic DNA isolated from Sunhwanogdu (VC1973A) and Kyunggijaerae#5 (V2984) leaves in the V1 growth stage. We utilized the Illumina Hiseq2000 and obtained ~209 million 101 bp reads from VC1973A and ~179 million 101 bp reads from V2984 (Supplementary Table S1). Reads were mapped to the mungbean reference genome (VC1973A) using Bismark software^2^,^12^, and binomial test was applied to obtain reliable calling of methylated cytosines, using unmethylated chloroplast genome as a control^6^ (Fig. 1A). We also performed RNAseq *via* Illumina Hiseq2000 on three biological replicates of leaf mRNA samples from both cultivars at the V1 growth stage, in order to understand the effect of DNA methylation on gene expression (Supplementary Table S2). A total of ~240 million and ~215 million reads for VC1973A and V2984, respectively, were mapped to the reference genome, and gene expression was quantified for both accessions.

### Genome methylation profiles

The VC1973A methylome contains 7,804,417 methylated CGs (mCG, 58.9% of all CGs), 9,092,603 mCHGs (51.5% of all CHGs), and 20,106,381 mCHHs (17.9% of all CHHs) (Fig. 1B and Supplementary Table S3). The total proportion of methylated cytosines was found to be greater than that of *Arabidopsis thaliana*^3^, but similar to that of *Glycine max*^6^. Notably, the proportion of mCHH sites was the highest in mungbean, a feature that distinguishes it from *A. thaliana* and *G. max,* which have mCG as the predominant sites^3^,^6^ (Fig. 1B). It is unclear why mCHH sites were increased in the mungbean genome; however, common bean also has increased mCHH sites, suggesting that the mCHH elevation occurred before the split of *Phaseolus* and *Vigna*^13^. The sequences flanking the cytosine methylation sites were random, although we found a higher tendency for adenine (A) or thymine (T) to be found there compared to the other bases, within the sequence contexts of mCHG and mCHH (Fig. 1C).

### Methylation profile of genes

To understand the distribution of DNA methylation around genes, we calculated the weighted methylation levels^14^. The distribution of the weighted methylation levels of whole genes in the mungbean genome showed different patterns depending on the cytosine contexts (Fig. 1D). That is, mCG and mCHG displayed two distinct peaks, representing the low- (~10%) and high-level (~80%) methylated genes. However, mCHH showed a narrow spectrum of methylation levels for all affected genes. This is possibly because the fraction of mCHHs to total CHHs is lower than the fractions of mCGs and mCHGs to their corresponding total cytosine contexts, even though a large number of mCHH methylations were detected.

The methylation levels of genes and their upstream and downstream regions showed distributions consistent with those detected in *A. thaliana* and *G. max.* That is, a slight increase was observed at transcription start sites and in the middle of gene coding regions, and a steep increase was found in the upstream and downstream regions (Fig. 1E)^3^,^6^. Conversely, the methylation levels of transposable elements (TE), as well as their upstream and downstream regions, showed highly increased TE body methylation, as compared to the upstream and downstream regions (Supplementary Fig. S1). Assuming that the high proportion of TE activities are suppressed, as compared to genes, these results are consistent with the suggested role of DNA methylation in controlling gene expression.

From the DNA methylation landscape of long assembled linkage groups, we could observe increased methylation counts in heterochromatin regions and decreased methylation counts in centromeric regions, which are consistent with observations from previous studies^3^, while the shorter assembled linkage groups showed truncated versions of the same pattern, possibly due to the incomplete assembly of the reference genome (Fig. 1F and Supplementary Fig. S2). Furthermore, the largest portion of DNA methylations occurred in intergenic regions, and the frequencies of mCGs, mCHGs, and mCHHs are maintained in untranslated regions (UTR), coding sequences (CDS), introns, 1 Kb upstream sequences, and intergenic regions. Within genic regions, the introns and exons contained more methylation than the 5′ and 3′ UTR regions (Supplementary Fig. S3).

### Association between gene methylation and expression

Using the quantified RNAseq expression data from the leaf tissue of VC1973A, we assessed the effect of DNA methylation on gene expression. We observed that for mCG and mCHG, the genes in the highly methylated peak (~80% regions) were rarely expressed compared to the low methylated (<10%) genes. Additionally, the genes that were methylated >5% by mCHH showed low expression levels (Supplementary Fig. S4). Overall, we found a negative correlation between gene body methylation (for mCG, mCHG, and mCHH) and gene expression level (Supplementary Fig. S5A). Within this group, we tested the effect of exon methylation on gene expression using genes that lack intron methylations. Conversely, the effect of intron methylation was determined using genes without exon methylations (Supplementary Figs S5B and S5C). From this analysis, we found that exon methylation affected gene expression more strongly than intron methylation.

### Role of DNA methylation in the fate of duplicates

**Figure 2:**
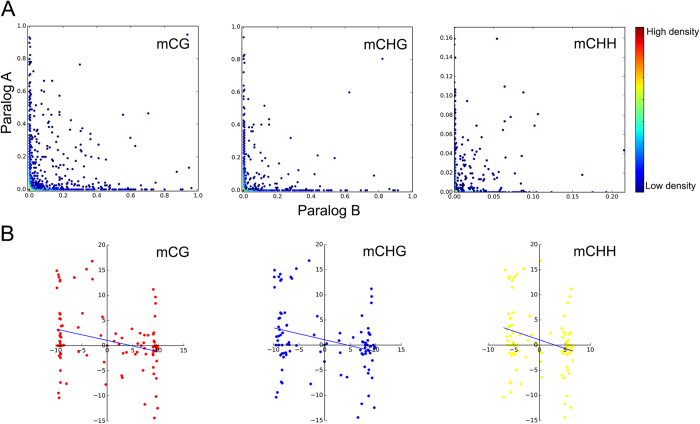
The effect of whole-genome duplication on DNA methylation. (**A**) Comparison of DNA methylation level between paralogous regions. (**B**) The correlation between gene methylation level and expression level; x- and y-axes depict the fold changes in gene methylation and expression levels, respectively.

As an ancient whole-genome duplication (WGD) in mungbean genome occurred nearly 59 million years ago (MYA)^2^, we assumed that the redundancy of the paralogs was well controlled by fractionation processes during this long evolutionary period^15^. We found that all types of DNA methylations were mostly differentiated between paralog A and paralog B, when the methylation states of paralogs being plotted on, or near, the x- or y-axis (Fig. 2A). A negative relationship was also observed between the fold changes in methylation and gene expression levels between the paralogs (Fig. 2B). This global trend between gene methylation and expression suggests that DNA methylation contributed to gene dosage control after the duplication. Cases that show a positive relationship between these measures implicate other factors in controlling gene expression. We further tested high copy number (7–8 copies) gene families that would have gone through duplication processes, such as large-scale and small-scale duplications (Supplementary Fig. S6). We observed that the extent of DNA methylation occurred differently for each gene family; however, the expression levels of genes within each family were not associated with DNA methylation levels. The gene expression levels within each family were highly variable, despite the consistent levels of DNA methylation. For example, the RPS2 gene family showed a very low level of DNA methylation, as well as very low gene expression levels. This suggests there are factors other than methylation level that control the expression of the high copy number genes.

### Comparison of DNA methylation states in mungbean cultivars

**Figure 3:**
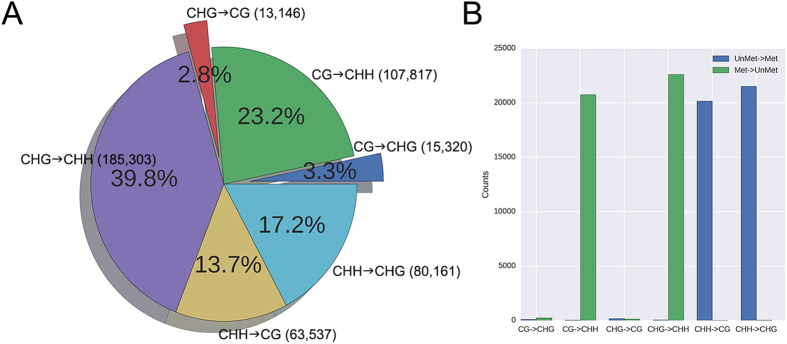
SNPs found in VC1973A versus V2984 that affect cytosine contexts. (**A**) Classification of SNPs found in VC1973A and V2984 in regards to cytosine context. (**B**) DNA methylation changes along with SNPs found in VC1973A and V2984.

We next compared the DNA methylation states of VC1973A and V2984. Of the commonly called 114,679,466 cytosine contexts, we found 85,832 sites that are differentially methylated (Fisher’s exact test, *P-*value < 0.01). It is possible that single nucleotide polymorphisms (SNPs) between the two cultivars affect DNA methylation levels by changing the cytosine context. Therefore, we surveyed the SNPs found in VC1973A versus V2984, using previously generated resequencing data^2^ to observe their effect on the DNA methylation status (CG<->CHG, CHG<->CHH, and CG<->CHH). The BSseq reads of V2984 were mapped to V2984 sequences that were constructed by substituting the reference genome with the SNPs. We found that a total of 465,284 SNPs changed the cytosine context. Among them, CHG->CHH SNPs were predominant, and CHG->CG SNPs were minor (Fig. 3A). There were also cases where the DNA methylation status was changed along with the SNPs. Interestingly, the interchange of methylation and un-methylation between VC1973A and V2984 was highly related to the cytosine context transition from/to CHH (Fig. 3B).

### Comparison of gene methylation states in mungbean cultivars

**Figure 4:**
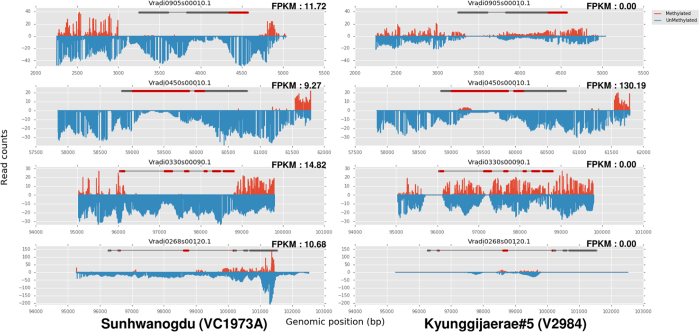
Comparison of the gene methylation and gene expression levels of four specific genes in VC1973A and V2984.

**Figure 5:**
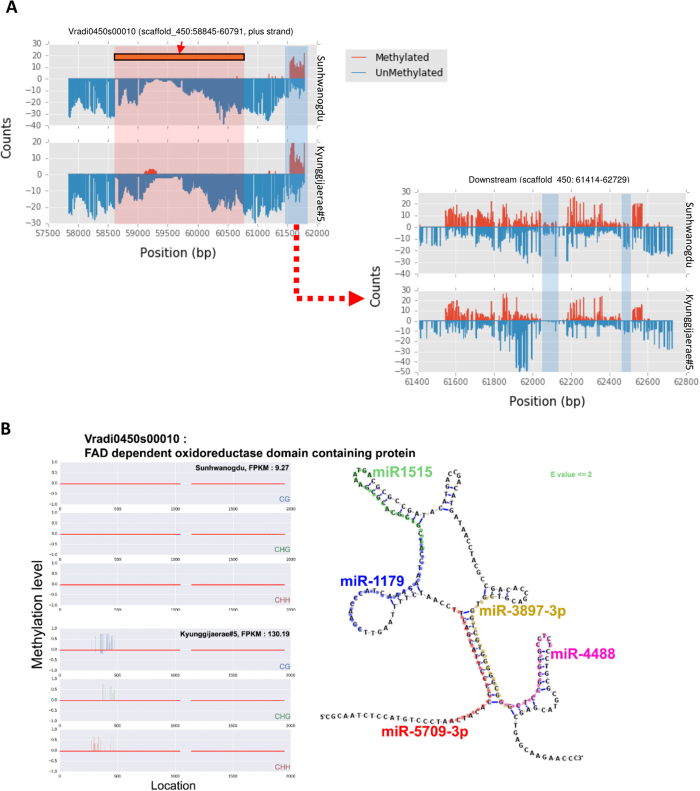
A case of increased gene expression with partial gene methylation in V2984 versus VC1973A. (**A**) DMR in downstream regions of the gene, (**B**) the partially methylated regions display secondary structure (right panel) and show homology with known miRNAs.

The differences in methylation status may lead to the varied gene expression levels between cultivars. Among VC1973A and V2984, we found that 3891, 2148, and 1703 genes were differentially methylated in the mCG, mCHG, and mCHH contexts, respectively. To see if differing methylation levels could explain the differentially expressed genes (DEG) between the two accessions, we compared gene expression levels from the RNAseq data using Cuffdiff software^16^. By analyzing the leaf transcriptomes of VC1973A and V2984 with stringent thresholds in the RNAseq analysis pipeline, a total of 29 DEGs were observed between the two cultivars. Among these DEGs, we were able to retrieve four cases where DNA methylation possibly affected gene expression (Fig. 4 and Supplementary Table S4). Vradi0905s00010, Vradi0330s00090, and Vradi0268s00120 were completely silenced and highly methylated within exon regions in V2984. Exceptionally, Vradi0450s00010 was partially methylated at the first exon in V2984; however, gene expression in this cultivar was highly increased, as compared to the VC1973A. Additionally, we surveyed differentially methylated regions (DMR) (Supplementary Table S5) to observe the difference of DNA methylations on the regulatory regions in genome which can control gene expression levels. The DMR in downstream regions of Vradi0450s00010 of VC1973A was more methylated than V2984 (Fisher’s exact test, *P value* < 0.001) suggesting that DNA methylation on possible downstream regulatory region may suppress the expression of this gene in VC1973A (Fig. 5A). Moreover, we suspect that DNA methylation may suppress the microRNAs (miRNAs) that regulate the corresponding gene. Hence, we surveyed the secondary structure of the methylated exon of Vradi0450s00010 and found possible hairpin structures (Fig. 5B). These DNA sequences also have homology to known miRNA sequences.

## Discussion

DNA methylation that occurs *via* known pathways in the CG, CHG, and CHH contexts is a type of epigenetic gene regulation^7^. DNA methylations across the whole mungbean genome were therefore surveyed to understand the role of DNA methylation in gene and genome evolution, and to elucidate their possible contribution to phenotypic differences, even between neighboring cultivars.

Here, we found that in the mungbean genome, the overall proportion of mCHH DNA methylation was the highest compared to the other possible methylation sites examined. This is quite different than what has been previously reported in the model plants, *Arabidopsis* and soybean^3^,^6^, where mCG is the most common type. Interestingly, the common bean, a species closely related to the mungbean, also contained the highest proportion of cytosine methylation in the CHH context^13^. The mungbean and the common bean are thought to have diverged ~8.0 million years ago (Ma), while the soybean is a relatively distantly related legume species that is believed to have diverged from the mungbean ~19.2 Ma^17^. From their speciation history, we postulate that an increased activity of the mCHH pathway in the common ancestor between the common bean and the mungbean might have occurred, and thus, the mCHH enriched DNA methylation traces are shared in both the mungbean and common bean genomes.

The gene content within a given genome can be increased by several types of duplication events, such as whole-genome duplication, segmental duplication, and tandem duplication^15^. A whole-genome duplication at around ~59 Ma in the mungbean genome resulted in the gene redundancy that manifests as many synteny blocks within the genome^2^. Our results suggest that DNA methylation is likely to contribute to gene expression control of the redundant genes within synteny blocks. However, genes with high copy number that arose from small-scale duplication events, such as tandem duplications, are not likely to be controlled by DNA methylation as they show differential gene expression regardless of the level of gene methylation (Supplementary Fig. S6). We propose two possible reasons for this; firstly, other factors, such as transcription factors or miRNAs, may be the major means for controlling the expression of genes that duplicate multiple times at a fast rate. Secondly, we suspect that certain genes are prone to be duplicated in small-scale duplications, and previous research indicates that the genes in these small-scale duplications are usually involved in the stress-response^18^. Hence expression of these genes would be more sensitive to be controlled by environmental factors, rather than changes within the cell.

The dependency of DNA methylation on genetic variants, such as those resulting from SNPs, allowed us to classify the epialleles into three major groups: obligate epialleles, pure epialleles, and facilitated epialleles^9^. Obligate epialleles are highly dependent on underlying genetic variations, and thus SNPs that change the cytosine context would change the state of DNA methylation and might consequently alter gene regulation. We listed the SNPs found in VC1973A versus V2984 that change the cytosine contexts and found that variations from/to CHH highly affect the DNA methylation status, suggesting the importance of the CHH context for obligate epialleles in the mungbean genome. We propose that these SNPs that change the CHH context should receive more attention for functional assay or in molecular breeding. In addition to these SNPs, VC1973A and V2984 also showed variations in DNA methylation. We assessed the possible consequences of these variations using an RNAseq approach and found an interesting case, in which a partially methylated coding sequence in V2984 showed increased gene expression compared to VC1973A, which has no DNA methylation in the coding sequence. Interestingly, the methylated part contained sequence similarity to previously reported miRNAs and was predicted to form secondary structures. Moreover, VC1973A contained more methylated blocks than V2984 in the downstream region of the gene, which may also control the mRNA expression. This suggests that partial methylation on the coding sequences with miRNA traces and the downstream sequences might contribute to the regulation of gene expression, which need further experimental validation.

Our BSseq analysis of the mungbean genome has expanded the understanding of genome-wide gene family regulation by DNA methylation. The notable feature of abundant mCHH traces in mungbean, and the frequent variations between cultivars as obligate epialleles, will also provide information that may prove valuable towards the goal of unraveling the role of *de novo* DNA methylation, and the consequent phenotypic variations within the mungbean germplasm.

## Methods

### Whole-genome bisulfite sequencing

To identify methylated genomic regions, we implemented bisulfite sequencing, or BSseq. Young leaves from VC1973A and V2984 were collected in triplicate at the third growth stage. DNA was extracted using the cetyltrimethyl ammonium bromide method (Gelvin and Schilperoot 1995), bisulfite treatment was performed, and DNA sequencing libraries were constructed for both VC1973A and V2984 samples. Briefly, input gDNA (5 μg) was fragmented by Covaris shearing (Covaris S2 series from Covaris Inc. [Woburn, MA, USA]). The fragments were blunt-ended and phosphorylated, and a single ‘A’ nucleotide was added to the 3’ ends in preparation for ligation to a methylated adapter that has a single-base ‘T’ overhang, using the TruSeq DNA library preparation kit provided by Illumina (Illumina, San Diego, CA, USA). The ligation products were then purified and size-selected by agarose gel electrophoresis. Size-selected DNA was subsequently bisulfite-treated twice and purified using procedures that were adapted from the EpiTect Bisulfite Kit (QIAGEN, Valencia, CA). The bisulfite-treated DNA was subsequently PCR-amplified to enrich for fragments that have adapters on both ends. The final purified product was then quantified using qPCR, according to the qPCR Quantification Protocol Guide and qualified using the Agilent Technologies 2100 Bioanalyzer (Agilent Technologies, Palo Alto CA, USA). BSseq libraries were then sequenced using the HiSeq™ 2000 platform (Illumina, San Diego, CA USA) for 101 cycles.

### RNA sequencing

We extracted RNA from three replicates of each VC1973A and V2984 leaf sample that was analyzed by BSseq (described above). RNA samples were validated using a 2100 bioanalyzer RNA 6000 NANO chip, and DNA samples were validated with Picogreen (Invitrogen, cat. #P7589). We selected high quality DNA and RNA pairs for each cultivar in triplicate. RNAseq libraries were constructed using the Illumina TruSeq Kit (Illumina, San Diego, CA), following the manufacturer’s guidelines.

### Sequencing analysis

The sodium bisulfite non-conversion rate was calculated as percentage of methylcytosines aligned with the unmethylated chloroplast genome (Mungbean chloroplast reference [KPS1 = VC1973A doi:10.1093/dnares/dsp025]). Reads produced from bisulfite sequencing were supplied to Bismark software^12^ with options; to build pre-converted forms of the reference, Bismark_genome_preparation module was used with bowtie 0.12.7, and we then aligned read files to the prepared reference genome with Bismark module under the default option. Among the genomic sites that were extracted using Bismark_methylation_extractor, we collected the sites for which we could detect >90% of the methylation level, with a supporting depth >10 to ensure reliability.

For the correction of methylation count, we used the mungbean chloroplast genome as negative control for the DNA methylation as it is known to highly unmethylated. Based on the read mapping status on the chloroplast genome, we could calculate the error rate of bisulfite sequencing method as 0.001114. Applying this error rate on the counts of methylated and unmethylated read supports on every cytosine contexts, we could determine the methylation status using binomial test. P values from the binomial test were converted into Q value for correct the significance level^19^. The methylated reads were corrected as follows; the supporting read numbers on the significant cytosine contexts were kept as they are, while the read numbers on the non-significant cytosine contexts were all regarded as number of unmethylated reads. The corrected supporting read numbers were used for the weighted methylation level of gene region^14^.

To retrieve DMRs, we retrieved the counts of the corrected methylated read supports within 200 bases window along with the chromosomes allowing 150 bases overlap. The windows containing more than 5 methylated reads were collected to define the candidate DMRs. This operation was done for both Sunhwanogdu (VC1973A) and Kyunggijaerae#5 (V2984). The final DMRs were determined if *Fisher’s exact test* shows significance (*P value* < 0.01) from the contingency table of the methylated and unmethylated counts of Sunhwanogdu and Kyunggijaerae 5 within each candidate DMR.

### Identification of duplicated genes and paralogs

With data >90% of the methylation level and supporting depth >10, paralogous genes were identified based on peptide homology using the NCBI blastp program (E-value ≤ 1e-5). The paralogous gene pairs were then clustered using MCScanX^20^.

## Additional Information

**How to cite this article**: Kang, Y. J. *et al*. Genome-wide DNA methylation profile in mungbean. *Sci. Rep.* **7**, 40503; doi: 10.1038/srep40503 (2017).

**Publisher's note:** Springer Nature remains neutral with regard to jurisdictional claims in published maps and institutional affiliations.

## Supplementary Material

Supplementary Information

Supplementary Table S5
