# Supplementary material for: Genome-wide DNA methylation profile in mungbean
Source: Sci Rep. 2017 Jan 13;7:40503. doi: 10.1038/srep40503 (PMC5233969; doi:10.1038/srep40503)
Supplement: Supplementary Information [file srep40503-s1.doc]

**Genome-wide DNA methylation profile in mungbean**

Yang Jae Kang^1^, Ahra Bae^1^, Sangrea Shim^1^, Taeyoung Lee^1^, Jayern Lee^1^, Dani Satyawan^1,2^, Moon Young Kim^1,3^ and Suk-Ha Lee^1,3,*^

*^1^Department of Plant Science and Research Institute of Agriculture and Life Sciences, College of Agriculture and Life Sciences, Seoul National University, Seoul 151-921, Korea*

*^2^Indonesian Center for Agricultural Biotechnology and Genetic Resources Research and Development, Bogor 16111, Indonesia*

*^3^Plant Genomics and Breeding Institute, Seoul National University, Seoul, 151-921, Korea*

*Correspondence should be addressed to Suk-Ha Lee (sukhalee@snu.ac.kr).

**Address:** Crop Genomics Lab. RN. 4105 Bldg. 200 CALS, Seoul National University, 1 Gwanak-ro, Gwanak-gu, Seoul 151-921, Korea

Tel: +8228804545

Fax: +8228774550

E-mail: [sukhalee@snu.ac.kr](mailto:sukhalee@snu.ac.kr).


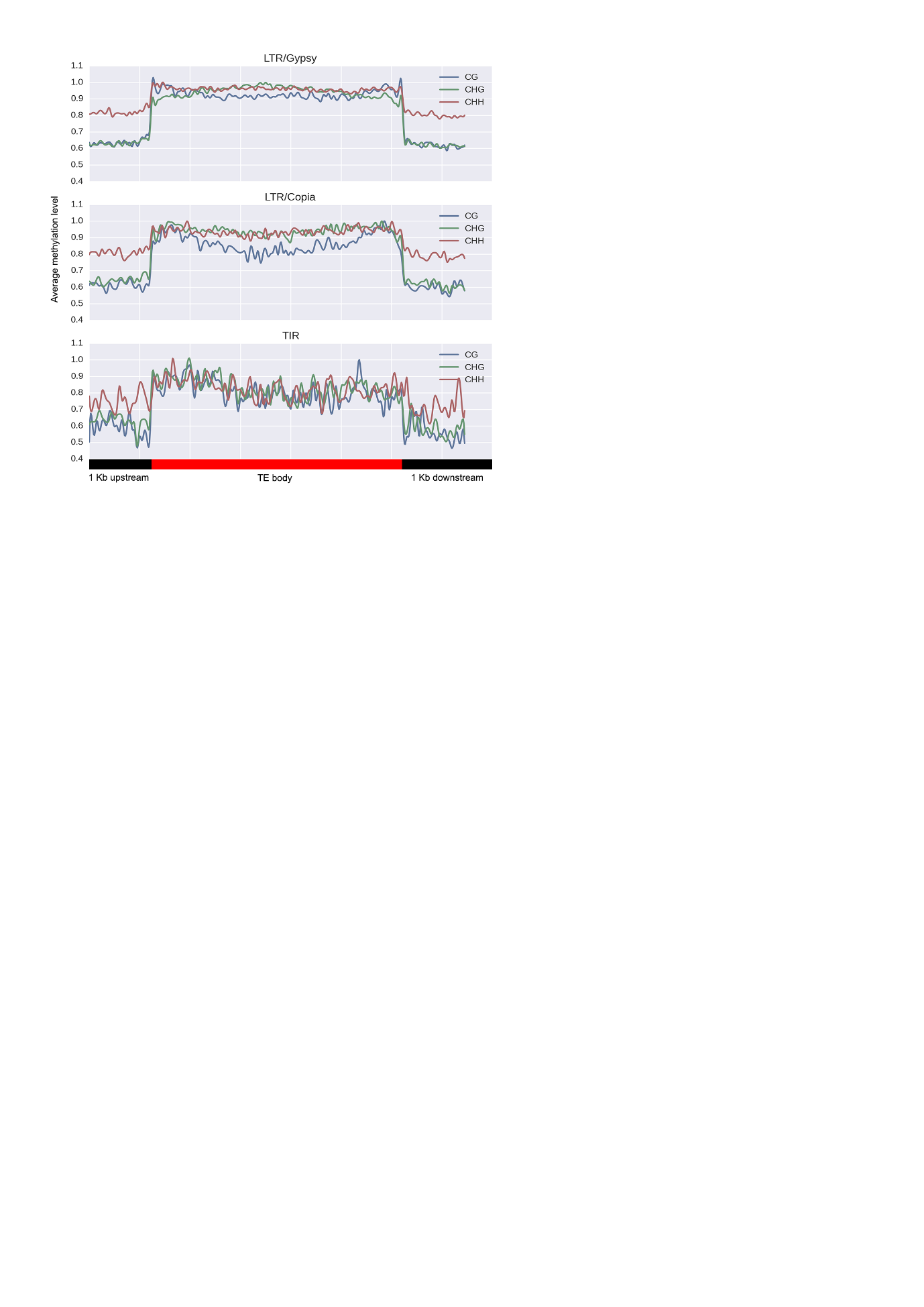


**Supplementary Figure 1.** The distribution of average methylation level across TE bodies and the up/down stream sequences of LTR/Gypsy, LTR/Copia, and TIR. Blue, green, red lines indicate CG, CHG, and CHH methylation, respectively.


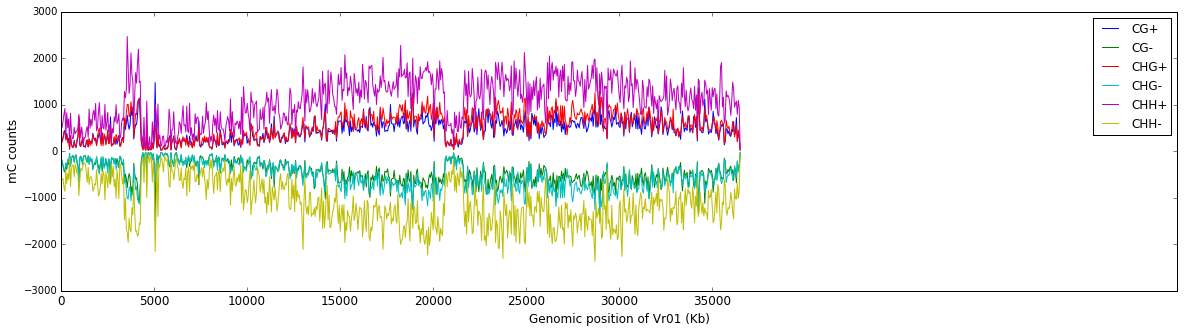


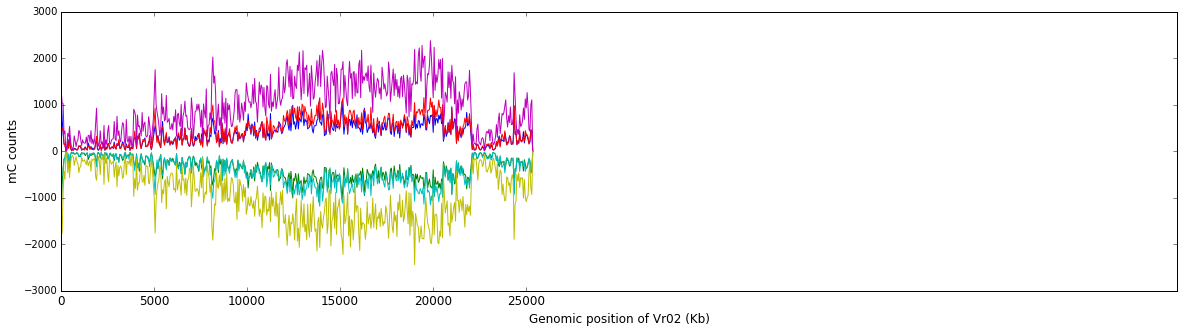


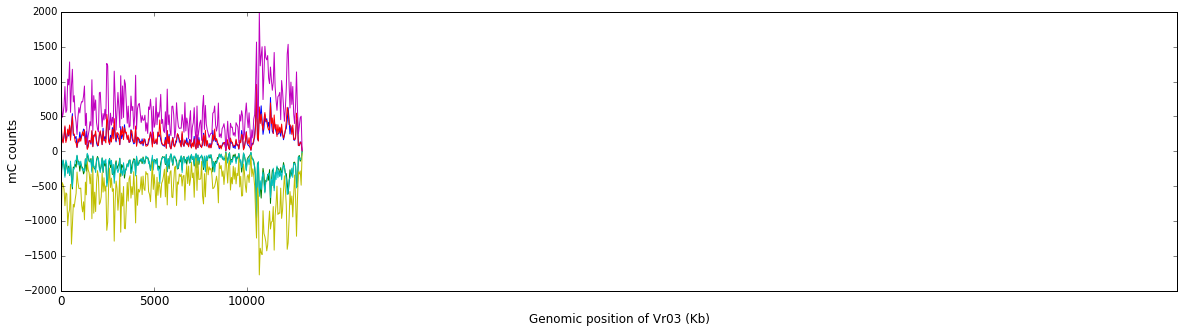


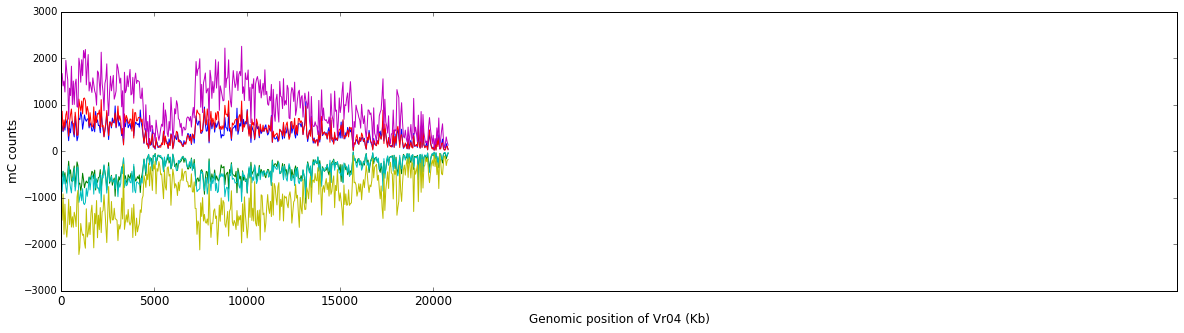


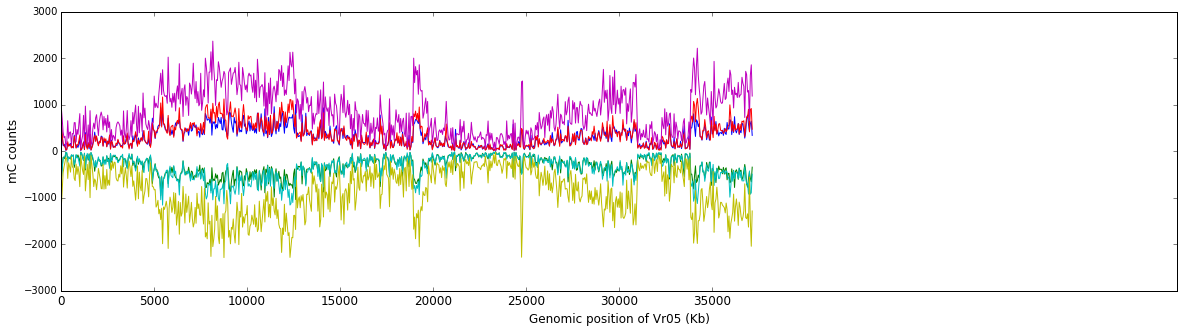


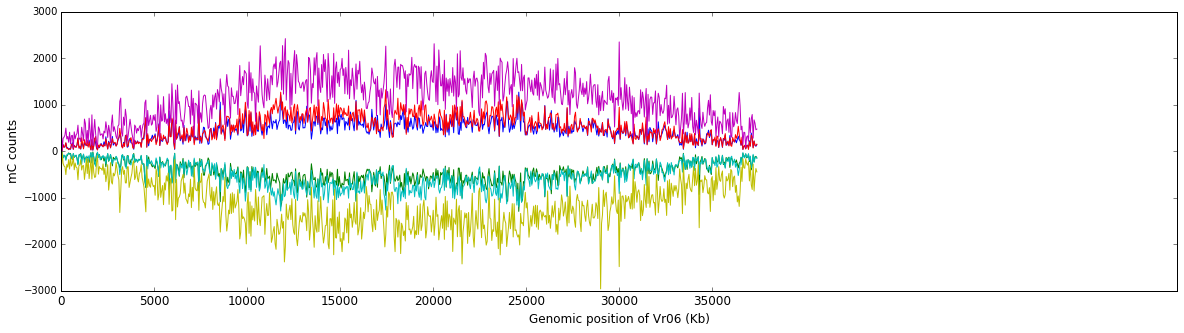


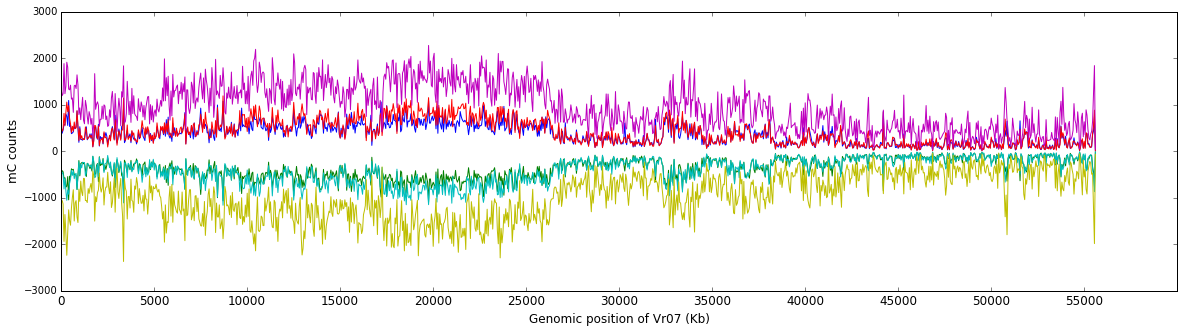


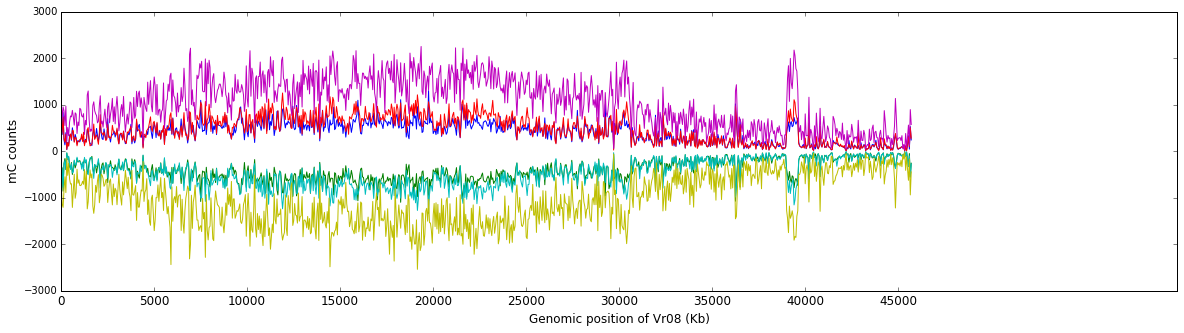


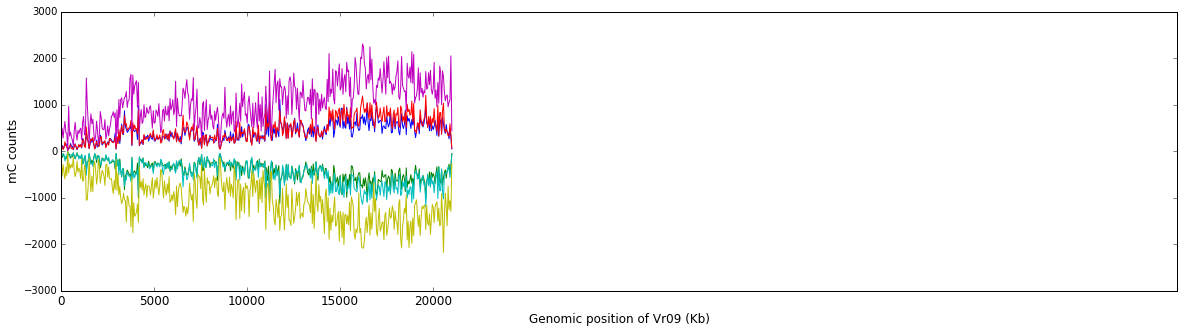


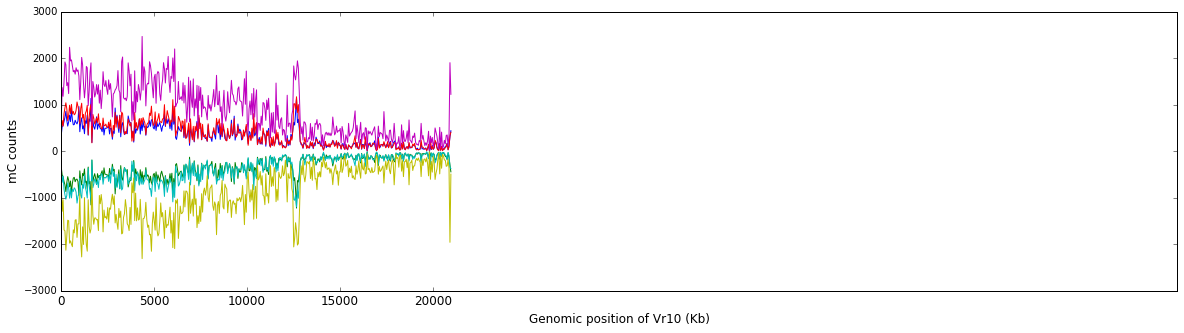


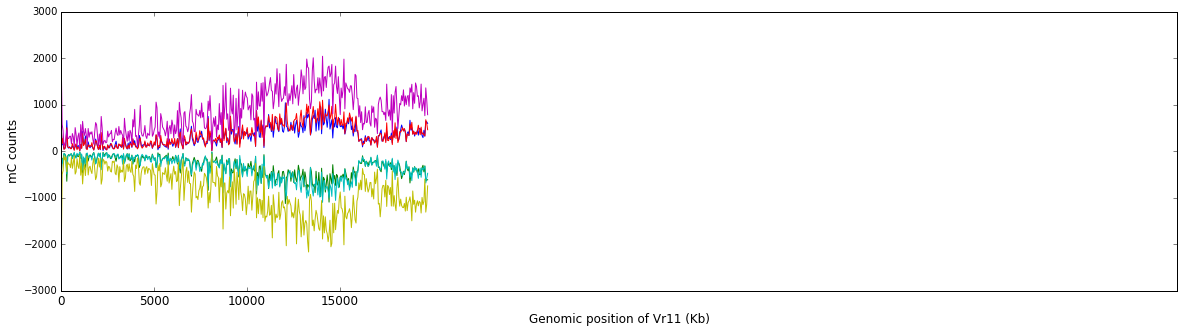

**Supplementary Figure 2.** DNA methylation landscape by each linkage group. Plus and minus strand methylations were displayed as plus and minus counts, respectively.


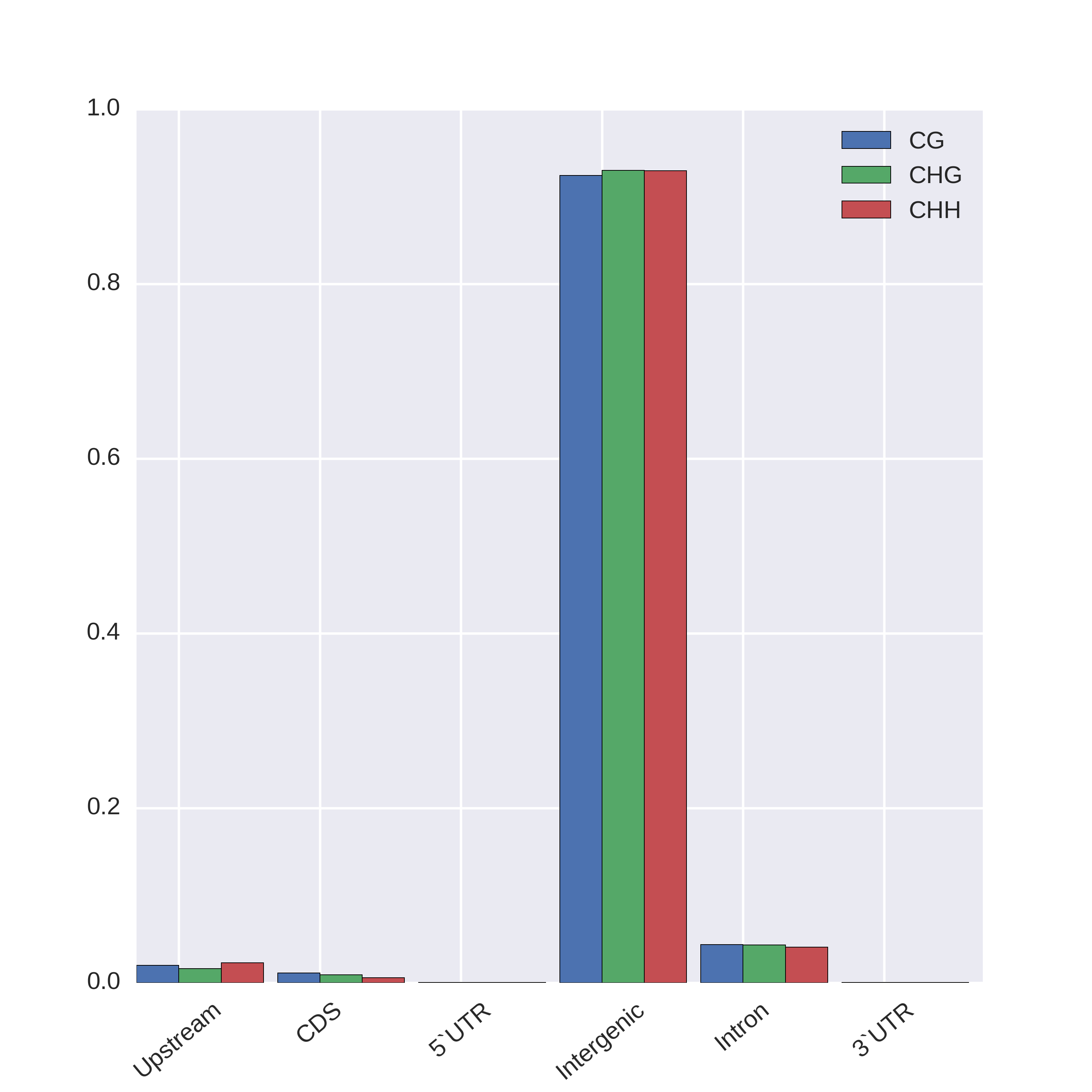


**Supplementary Figure 3.** Classification of mCG, mCHG, and mCHH sites according to mungbean gene model including 5’UTR, CDS, intron, 3’UTR, upstream regions and intergenic regions.


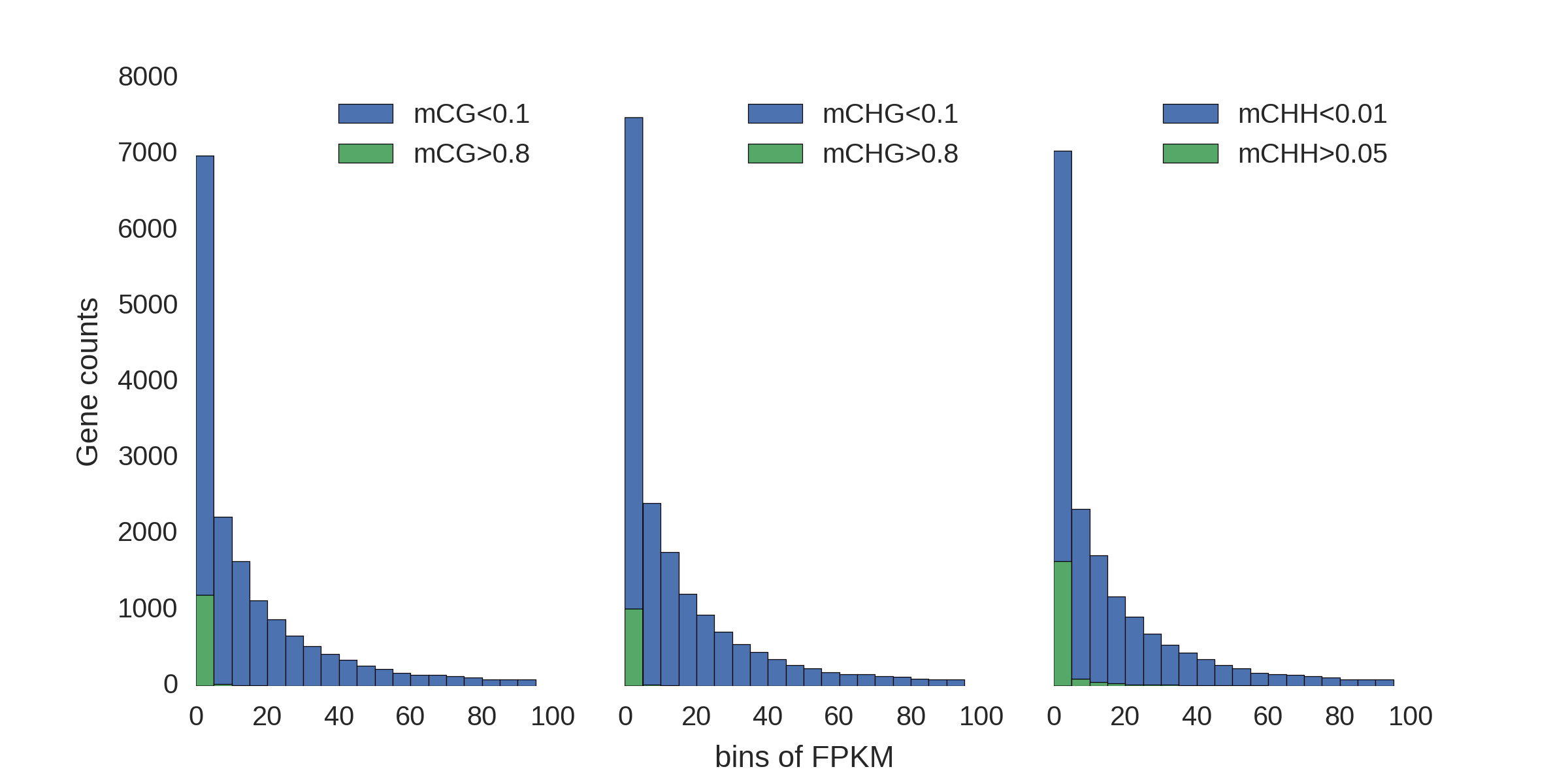


**Supplementary Figure 4**. Distribution of FPKM of high methylated genes and low methylated genes. The high methylation was defined over 80% for mCG and mCHG and over 5% for mCHH. The low methylation was defined less than 10% for mCG and mCHG and less than 1% for mCHH.


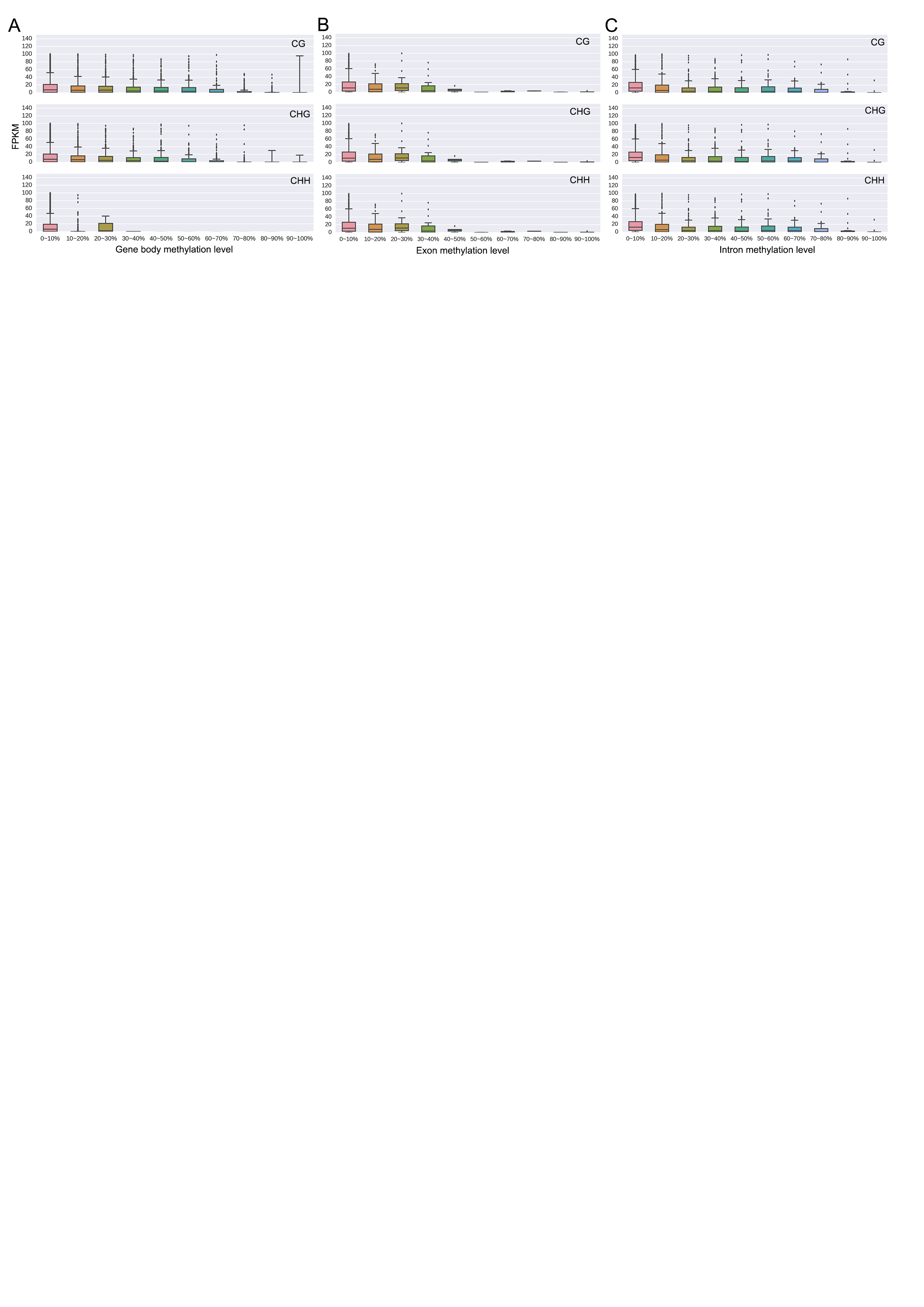


**Supplementary Figure 5.** The correlation between DNA methylation and gene expression level; A. correlation with gene body methylation, B. correlation with exon methylation, C. correlation with intron methylation. To understand the effect of exon and intron methylation to gene expression, we selected the genes with exon/intron methylation and without intron/exon methylation.


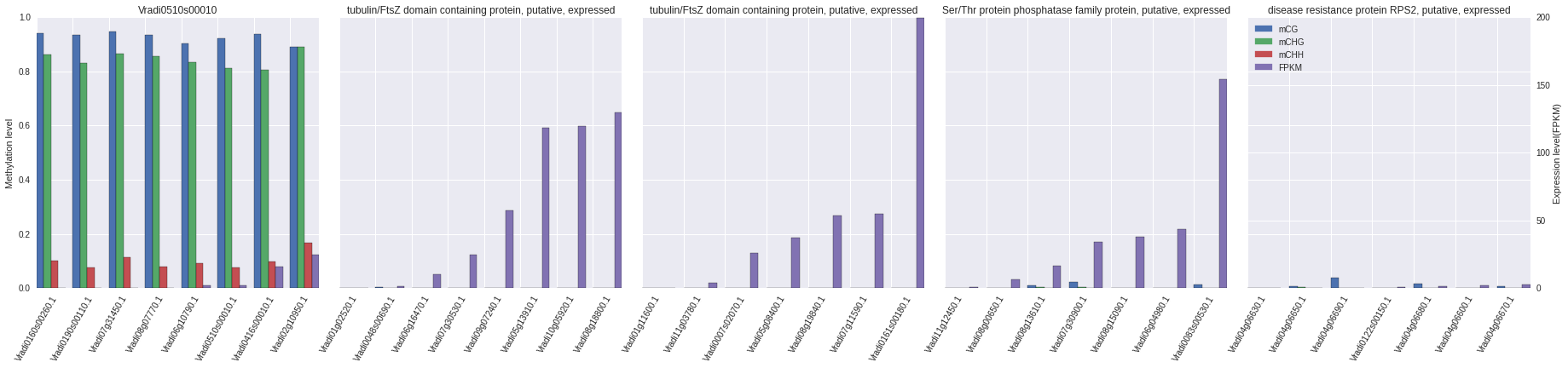


**Supplementary Figure 6**. Comparison of DNA methylation and gene expression level of high copy number gene families.

| **Supplementary Table 1.** BS-Seq information of VC1973A and V2984 | | | | | | |
| --- | --- | --- | --- | --- | --- | --- |
| Purpose | Acc no | Name | Platform | Library | Total reads | Sequencing depth^*^ |
| Bisulfite sequencing | VC1973A | Seonhwanogdu | Illumina Hiseq2000 | paired-end | 209,103,160 | 38.5 |
|  | V2984 | Kyung-Ki Jaerae#5 | Illumina Hiseq2000 | paired-end | 179,540,698 | 33.1 |
| * Sequencing depth were determined using estimate genome size using kmer analysis (Kang *et al*, 2014) | | | | | | |

| **Supplementary Table 2.** Summary of RNA seq result using Illumina Hiseq in paired end library | | | | |
| --- | --- | --- | --- | --- |
| Purpose | Cultivar | Tissue | Total reads | Total produced base |
| Gene expression quantification | VC1937A | Leaf – Rep. 1 | 65,254,568 | 6,590,711,368 |
|  |  | Leaf – Rep. 2 | 99,968,084 | 10,096,776,484 |
|  |  | Leaf – Rep. 3 | 74,902,964 | 7,565,199,364 |
|  |  | Total | 240,125,616 | 24,252,687,216 |
|  | V2984 | Leaf – Rep. 1 | 76,758,830 | 7,752,641,830 |
|  |  | Leaf – Rep. 2 | 82,690,052 | 8,351,695,252 |
|  |  | Leaf – Rep. 3 | 55,831,990 | 5,639,030,990 |
|  |  | Total | 215,280,872 | 21,743,368,072 |
|  |  |  |  |  |

| **Supplementary Table 3. Bismark bisulfite reads mapping results** | | |
| --- | --- | --- |
|  | VC1973A | V2984 |
| Sequence pairs analysed | 104551580 | 89770349 |
| Mapping efficiency | 74.10% | 65.80% |
| Total c analysed | 2189874117 | 1739218275 |
| mCpG | 107313781 | 81527806 |
| mCHG | 116306461 | 87313121 |
| mCHH | 95774535 | 63221509 |
| C to T CpG | 90990936 | 81923051 |
| C to T CHG | 146309856 | 123877007 |
| C to T CHH | 1633178548 | 1301355781 |
| methylC in CpG | 54.10% | 49.90% |
| methylC in CHG | 44.30% | 41.30% |
| methylC in CHH | 5.50% | 4.60% |
|  |  |  |

| **Supplementary Table 4.** Comparison of gene methylation and expression between Sunhwanogdu and Kyunggijaerae#5 | | | | | | | | |
| --- | --- | --- | --- | --- | --- | --- | --- | --- |
| Genename | Methylation level | | | | | | Gene expression (FPKM) | |
|  | Sunhwanogdu | | | Kyunggijaerae#5 | | | Sunhwanogdu | Kyunggijaerae#5 |
|  | CG | CHG | CHH | CG | CHG | CHH |  |  |
| Vradi0268s00120.1 | 0.40 | 0.47 | 0.04 | 0.88 | 0.71 | 0.11 | 10.68 | 0.00 |
| Vradi0330s00090.1 | 0.00 | 0.07 | 0.01 | 0.91 | 0.90 | 0.09 | 14.82 | 0.00 |
| Vradi0905s00010.1 | 0.00 | 0.00 | 0.00 | 0.94 | 0.85 | 0.10 | 11.72 | 0.00 |
| Vradi0450s00010.1 | 0.00 | 0.00 | 0.00 | 0.09 | 0.02 | 0.01 | 9.27 | 130.19 |
|  |  |  |  |  |  |  |  |  |
